# Postpartum depression and mother–offspring conflict over maternal investment

**DOI:** 10.1093/emph/eoaa049

**Published:** 2021-01-02

**Authors:** Annika Gunst, My Sundén, Riikka Korja, Amy M Boddy, Jennifer Kotler, E Juulia Paavonen, Henna-Maria Uusitupa, Linnea Karlsson, Hasse Karlsson, Jan Antfolk

**Affiliations:** 1 Department of Psychology, Åbo Akademi University, Tehtaankatu 2, Turku 20500, Finland; 2 Department of Psychology, University of Turku, Assistentinkatu 7, Turku 20014, Finland; 3 Department of Clinical Medicine, FinnBrain Birth Cohort Study, Turku Brain and Mind Center, University of Turku, Lemminkäisenkatu 3, Turku 20014, Finland; 4 Department of Anthropology, University of California, Santa Barbara, CA 93106 3210, USA; 5 Department of Organismic and Evolutionary Biology, Harvard University, 26 Oxford Street, Cambridge, MA 02138, USA; 6 Department of Psychology, Harvard University, 33 Kirkland Street, Cambridge, MA 02138, USA; 7 Department of Public Health Solutions, Finnish Institute for Health and Welfare, P.O. Box 30, 00271 Helsinki, Finland; 8 Centre for Population Health Research, Turku University Hospital and University of Turku, Kiinamyllynkatu 10, Turku 20520, Finland; 9 Pediatric Research Center, Helsinki University Hospital, P.O. Box 63, 00014 Helsinki, Finland; 10 Department of Psychiatry, Turku University Hospital and University of Turku, Lemminkäisenkatu 3, Turku 20014, Finland

**Keywords:** postpartum depression, mother–offspring conflict, infant night waking, maternal sleep, breastfeeding, fatigue

## Abstract

**Background and objectives:**

As the mother–offspring relationship is central to human reproduction, postpartum depression symptoms are difficult to explain in evolutionary terms. We proposed that postpartum depression might arise as a result of evolutionary mother–offspring conflict over maternal investment, and investigated the association between postpartum depression symptoms, infant night waking, maternal sleep disturbance and breastfeeding frequency.

**Methodology:**

We conducted a cross-sectional analysis using survey responses at 6 months postpartum from 1598 Finnish mothers. We hypothesized that infant night waking at 6 months postpartum would be associated with postpartum depression symptoms, and that this association would be mediated by maternal sleep disturbance and a higher breastfeeding frequency.

**Results:**

Infant night waking was moderately associated with postpartum depression symptoms, and this association was mediated by maternal sleep disturbance (*R*^2^=0.09). Contrary to our prediction, we found that increased breastfeeding was associated with less postpartum depression symptoms.

**Conclusions and implications:**

We conclude that postpartum depression symptoms might partly be the result of increased maternal fatigue stemming from high offspring demands on maternal investment, but that this is not due to the metabolic strain from increased breastfeeding. Studying postpartum depression from the mother–offspring conflict perspective can potentially improve our understanding of the involved behavioral processes of both mother and offspring, and allow interventions designed to benefit the well-being of both parties.

**Lay Summary:** We proposed that postpartum depression is due to an evolutionary conflict between mother and infant, where the infant tires the mother to delay the arrival of a sibling. We found a link between infant night waking and postpartum depression, mediated by the mother’s sleep, but not by breastfeeding frequency.

## INTRODUCTION

Postpartum depression (PPD) is defined as a depressive disorder, with an onset of symptoms (e.g. feelings of sadness and/or guilt, loss of pleasure and anxiety) during pregnancy or within the first year postpartum [1, 2]. As many as 15% of mothers suffer from PPD in the postpartum period, and many more report subclinical depressive symptoms, often referred to as the ‘baby blues’ [3, 4]. PPD has been observed in various cultural contexts [5], indicating that it is not only a Western phenomenon.

PPD can negatively affect cognitive functioning (e.g. impaired executive functioning and self-regulation) and parental behavior and, consequently, have an adverse effect on mother–child interactions and infant development [6]. At its worst, PPD can affect a mother’s ability to care for her child, or even lead a mother to actively harming herself and her infant, thereby dramatically decreasing her own and her infant’s fitness. Several studies report an association between PPD and increased infant mortality [7, 8].

The evolutionary approach to human behavior aims to identify psychological traits that have evolved as functional adaptations to recurring problems in human ancestral environments. As such, previous work theorizes that PPD may be an adaptive response that informs the mother that the costs of raising the offspring exceed the reproductive benefits. This work proposes that depressive symptoms may help the mother to negotiate greater levels of investments from others (i.e. social support), and sometimes decrease her investment in the infant (i.e. the defection hypothesis) [9]. Indeed, humans are cooperative breeders and have evolved in sociocultural contexts, in which critical social support for the mother during the perinatal period can be obtained [10]. PPD has also been suggested to result from recent shifts in human lifestyle, such as changes in diets, physical activity and family and social networks (i.e. the mismatch hypothesis) [11]. While PPD is observed broadly across cultural context, the rates and severity of PPD vary across cultures [12]. While there are insufficient prevalence estimates of PPD in small-scale subsistence societies, at least one recent study reported a high prevalence of PPD symptoms among Hadza foragers in Tanzania [13]. Recent meta-analyses include populations that vary in terms of urbanization and development. In these analyses, there is no clear evidence for PPD being a result of urbanization and/or industrialization [5]. Interestingly, PPD appears to be more prevalent in some rural communities (Bangladeshi [14], Amazonian [15] and Israeli Bedouin [16] mothers). These large meta-analyses do not, however, address the more intricate aspects needed to understand the development of PPD at the individual level, such as reduced social support.

Here, we propose and conduct initial testing for the theory that PPD symptoms might partly derive from evolutionary mother–offspring conflict over maternal investment. We believe that an evolutionary driver for this outcome results from evolutionary motivation by the infant to delay the arrival of a sibling—and thereby reduce sibling rivalry over maternal investment—by exhausting the mother. We highlight that the proposed theory is an attempt to provide an ultimate explanation for why PPD exists. We acknowledge that variation in several cultural practices likely also contributes to the variation in prevalence reported globally. 

### Infant night waking as an adaptive strategy

According to the theory of mother–offspring conflict, what is considered optimal maternal investment from the infant’s perspective (i.e. any investment of the mother that increases the infant’s chance of surviving) is higher than the optimal investment from the mother’s perspective (i.e. how costly the investment is to her) [17]. Inclusive fitness theory states that infants are selected to favor the birth and survival of future siblings, but not more than their own survival [18]. The introduction of siblings will decrease the mother’s investment of her limited resources in any one infant. By contrast, a mother will optimize her reproductive output by balancing her investment between all her current and future offspring. Accordingly, a ‘short’ interbirth interval is harmful for the infant (i.e. <2 years) [19], as it results in earlier weaning, may indicate a competitor sibling on the way, and increases the risk of infant mortality. For instance, infant mortality of! Kung hunter-gatherers has been reported to drop from over 70% to around 10% when extending the interbirth interval from 2 to 4 years [20] (whereas the average interbirth interval for humans in foraging populations has been estimated at 3.34 years) [21]. However, a ‘long’ interbirth interval is not beneficial for the mother as it might result in fewer children overall, thereby decreasing her lifetime reproductive output. Consequently, offspring are selected to favor longer interbirth intervals than what is optimal for maternal fitness [22].

Based on these conflicting fitness optima between mother and offspring, night waking to breastfeed has been proposed to be adaptive for the infant by expanding the interbirth interval of the mother [20, 23]. An important proximate cause of the length of the interbirth interval in naturally fertile populations is the duration of lactational amenorrhea [24]. Indeed, frequent breastfeeding, and especially breastfeeding at night, upholds lactational amenorrhea [25]. Interestingly, infant night waking is common throughout the first years of life, regardless of calories consumed during the day [26, 27].

### PPD as a consequence of fatigue

**Figure 1.**
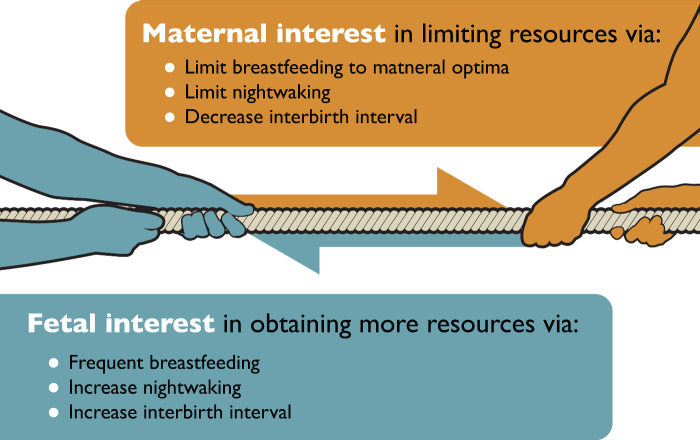
Mother–offspring conflict illustrated as a tug-of-war analogy in the context of postpartum depression. It is in the mother’s interest to limit resources to distribute to future offspring. In contrast, the offspring’s interest is to obtain more resources than the optimal investment for the mother to provide. The mother–fetal dyad is a co-evolved unit, and as such, there is a perceived ‘balanced’ outcome from these divergent genetic interests. Imbalance in this system (i.e. the fetus extracts more resources than the mother can provide), may lead to postpartum depression symptoms, which has been proposed to be an adaptive response to signal for help.

Infant night waking might also extend the interbirth interval by contributing to maternal fatigue (see Fig. 1). By tiring the mother, the infant could help delay the arrival of a sibling, as exhausted mothers might not have the energy needed to conceive and gestate another child (for instance, by decreasing the mother’s sex drive [28]; or by reducing her fertility by hypothalamic-pituitary-adrenal activation) [29]. Indeed, infant night waking and maternal fatigue in the postpartum period have repeatedly been associated with PPD [30–33]. In a prospective study of mothers during the first two years postpartum [34], infant night waking increased subsequent PPD symptoms. PPD could, in other words, represent a maternal response to counter the infant’s otherwise ‘functional’ strategy to tire the mother.

Frequent breastfeeding in itself subjects the mother to a considerable metabolic strain by depleting energy resources [35]. This could, at least partly, explain why some mothers experience fatigue in the postpartum period, and this fatigue could be amplified depending on milk quality and quantity. Energy levels postpartum are also affected by the sleep quality of the mother. A recent study [36] found that maternal sleep satisfaction and duration sharply declines after childbirth and is at its lowest point the first 3 months postpartum, after which neither sleep duration nor satisfaction fully recovers for several years. In general, sleeping problems have been strongly associated with depressive symptoms [37], and are common features of depression [1]. Some studies have reported that the relationship between infant sleep problems and maternal mental health issues diminishes when controlling for maternal sleep quality [30, 38], indirectly indicating that maternal sleep quality mediates the relationship between infant sleep quality and PPD. However, the association between infant night waking, maternal fatigue through breastfeeding, sleep disturbance and PPD symptoms, has to our knowledge not been investigated from the perspective of mother–offspring conflict.

### The current study

Our aim was to investigate the association between infant night waking and PPD symptoms at 6 months postpartum, from the evolutionary perspective of mother–offspring conflict. We studied whether an association between infant night waking and PPD symptoms would be mediated by maternal sleep disturbance and/or breastfeeding frequency. Based on previous research and theoretical considerations, we predicted that (i) infant night waking at 6 months postpartum would be associated with PPD symptoms, and that this association would be mediated by (ii) maternal sleep disturbance and (iii) increased breastfeeding frequency. We further explored these relationships taking into account the infant’s sex, infant formula use, co-sleeping and parity. We also expected the association between infant night waking and PPD symptoms to be weaker for those using infant formula, as these mothers would be less metabolically strained and as potential other caregivers could take care of these feeds.

## METHODOLOGY

### Ethical statement

Data were obtained from the FinnBrain birth cohort study [39]. The Ethical Board for the Hospital District of Southwest Finland provided an ethical statement in favor of the FinnBrain project (20 June 2011, no. 6/2011). This study is in line with the informed consent forms signed by the mother, the father and by the legal guardians of the focal child.

### Participants

The original sample included 3838 Finnish children and their families, who had been followed since the first ultrasound screening at gestational week 14 until 2 years of age. From the data available, we selected variables that measured infant night waking, maternal sleep disturbance, breastfeeding frequency and PPD symptoms. This study was cross-sectional. As our measures were taken 6 months postpartum, confounding factors, such as sleep-onset association difficulties (which often emerge at a later age) [40], were all but avoided. Participants who had not responded to all study variables at the 6-month postpartum timepoint (*n* = 2240) were excluded. Thus, the final sample included 1598 mothers (*M*_age_ = 30.7 years; *SD* = 4.7, range 18–44). Of the included mothers, 52% (*n* = 826) were primiparous, and 88% (*n* = 1403) reported that they were married or co-habiting. Of the infants, 53% (*n* = 853) were male and 47% (*n* = 745) were female. Detailed participant demographics are presented in Table 1.

**Table 1.** Participant demographics

	*n*	%
Infant’s sex		
Male	853	53.4
Female	745	46.6
Infant’s sleeping arrangement		
Bed in separate room	208	13.0
Bed in parents’ room	1016	63.6
In parents’ bed	259	16.2
Bed in siblings’ room	107	6.7
Infant formula use (sometimes or regularly)		
Yes	1048	65.6
No	524	32.8
Mother’s parity		
Primiparous	826	51.7
Multiparous	706	44.2
Mother’s relationship status^a^		
Married or co-habiting	1403	87.8
In a relationship	75	4.7
Divorced	5	0.3
Widowed	0	0
Registered partnership	4	0.3
No relationship	10	0.6
Mother’s education level^a^		
Part of compulsory school	0	0
Compulsory school	23	1.4
Vocational course/apprenticeship	16	1.0
Vocational school	232	14.5
High school graduate	199	12.5
Bachelor’s degree (applied)	437	27.3
Bachelor’s degree (university)	94	5.9
Master’s degree	433	27.1
Licentiate/doctorate degree	83	5.2
Other	22	1.4
Mother’s monthly income^a^		
<500€	151	9.4
501–1000€	169	10.6
1001–1500€	229	14.3
1501–2000€	504	31.5
2001–2500€	313	19.6
2501–3000€	101	6.3
3001–3500€	49	3.1
3501–4000€	11	0.7
>4000€	11	0.7

Note that demographic variables include missing data. Hence, the numbers do not always add up to *n* = 1598 (maximum missing for separate variable 6.3%).

a Assessed 1 month postpartum.

## MEASURES

In this study, we modeled associations between latent factors of infant night waking (initially including four indicators), maternal sleep disturbance (three indicators), breastfeeding frequency (two indicators) and PPD symptoms (10 indicators), based on survey data collected from the mothers at 6 months postpartum. The indicators (i.e. items) included are described below.

### Infant night waking

Information about infant night waking was obtained using the Brief Infant Sleep Questionnaire (BISQ) [41]. The BISQ is a 12-item questionnaire that includes questions about the infant’s sleep during the past week. The original scale has demonstrated sound psychometric properties in different populations [35, 42]. In this study, we used items 5, 7 and 8, in which the mother rates the average amount of nighttime sleep in hours, night awakenings, and hours spent awake during the night for the infant. Moreover, we used item 12, in which the mother rates their infant’s sleep problems on a 3-point Likert scale (0–2). In this study, all items were coded so that higher scores indicated more infant night waking.

### Maternal sleep disturbance

Information about maternal sleep disturbance was obtained using the Basic Nordic Sleep Questionnaire (BNSQ) [43]. The BNSQ is a 27-item self-report questionnaire inquiring about sleep-related behaviors and disturbances during the past 3 months. The BNSQ has been used in a variety of studies conducted in the Nordic countries [43]. We included item 4, in which the mother rates the average number of night awakenings on a 5-point Likert scale (1–5), as well as item 12, in which the mother estimates her average amount of nighttime sleep in hours. We also used a composite variable for daytime sleepiness that was created by summing the scores on items 8–11, all of which were 5-point Likert scale items (1–5). Thus, the possible range of the composite variable was 4–20 points. In this study, all items were coded so that higher scores indicated more maternal sleep disturbance.

### Breastfeeding frequency

Three variables were selected from the questions designed for the FinnBrain birth cohort study: (i) a variable measuring the total breastfeeding duration in days, from birth to 6 months of age. We converted this variable from measuring breastfeeding duration in days to measuring breastfeeding duration in months. The maximum value of this variable was 6 months. Note, however, that some mothers likely continued breastfeeding beyond the 6-month measurement; (ii) a variable measuring how many times during a 24-h day the infant breastfed on average, given that the infant was exclusively breastfeeding. This variable included only participants who had responded that their infant was exclusively breastfeeding; (iii) a variable measuring how many times during a 24-h day the infant breastfed on average, given that the infant was partially breastfeeding but also drinking infant formula or eating solid foods. This variable included only participants who had responded that their infant was partially breastfeeding. In the statistical analysis, variables 2 and 3 were combined into one variable. Participants who had ceased breastfeeding before 6 months postpartum were assigned the value zero on this variable. For both included variables, higher scores indicated more frequent breastfeeding.

### PPD symptoms

Information about PPD symptoms was obtained using the Edinburgh Postnatal Depression Scale (EPDS) [44]. The EPDS is a 10-item self-report questionnaire, designed to assess symptoms of PPD during the past week. The EPDS has demonstrated sound psychometric properties in different populations [45, 46]. In this study, we used all 10 items, scored on a 4-point Likert scale (0–3). All items were coded so that higher scores indicated more depressive symptoms. A cut-off indicating probable depression has been suggested at 12–13 points [47]. Here, we used a continuous measure of PPD ‘symptoms’ and not a categorical measure based on the diagnostic cut-off.

## STATISTICAL ANALYSES

We analyzed the data in *R* (version 3.5.0). To adjust for measurement error in the analyses, we conducted a structural regression model with latent factors, utilizing the ‘lavaan’ package. For the visualization of correlations between items, we utilized the ‘corrplot’ package. As some of the variables were ordered, we used the diagonally weighted least squares estimator.

## RESULTS

### Descriptive statistics

Descriptive statistics for the included variables are presented in online supplementary table 1. These variables were used as indicators when creating the latent variables in the structural regression model. In this sample, 812 (50.8%) infants were not breastfeeding.

### Correlations

**Figure 2.**
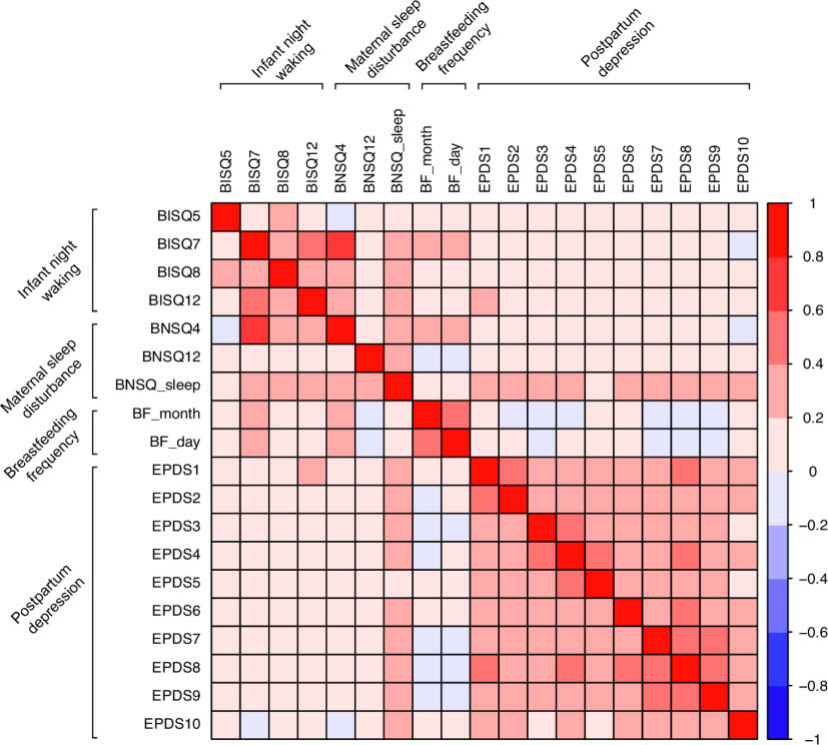
Zero-order Spearman correlations between the variables included as indicators in the latent factors. All correlations were significant at the *P* <0.001 level. BISQ, Brief Infant Sleep Questionnaire; BNSQ, Basic Nordic Sleep Questionnaire; BF, breastfeeding items (indicating breastfeeding duration in months and daily breastfeeding frequency); EPDS, Edinburgh Postnatal Depression Scale. The number after the abbreviation refers to the item number of said questionnaire.

Correlations between separate variables are illustrated in Fig. 2.

### Results from the structural regression model

#### Model specification

**Figure 3.**
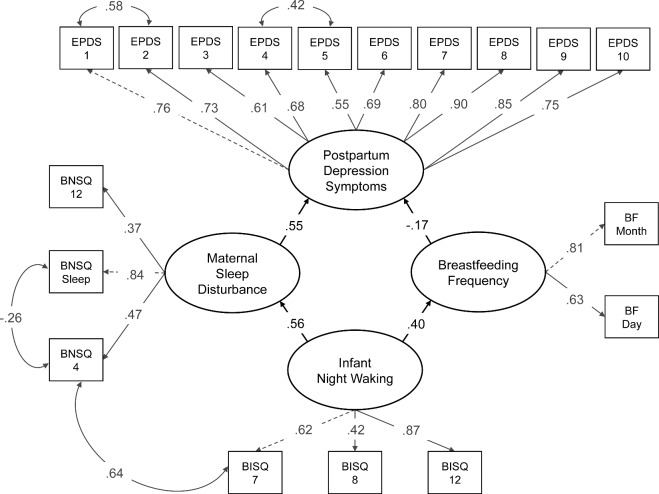
Standardized estimates for the adjusted structural regression model. Latent variables are depicted as ovals; observed variables as rectangles; regression coefficients as single-headed arrows; correlations as two-headed arrows. Indirect effects and residual errors are not shown. BISQ, Brief Infant Sleep Questionnaire; BNSQ, Basic Nordic Sleep Questionnaire; BF, breastfeeding items (indicating breastfeeding duration in months and daily breastfeeding frequency); EPDS, Edinburgh Postnatal Depression Scale.

First, we specified a model, which included four latent variables: ‘Infant Night Waking’ (four indicators), ‘Maternal Sleep Disturbance’ (three indicators), ‘Breastfeeding Frequency’ (two indicators) and ‘Postpartum Depression Symptoms’ (10 indicators). The assumption in our model specification was that infant night waking at 6 months postpartum would be associated with PPD symptoms, and that this association would be mediated through breastfeeding frequency and maternal sleep disturbance. As the initial model showed suboptimal fit [χ^2^(148) = 1742.994, *P* <0.001, CFI = 0.898, TLI = 0.882, RMSEA = 0.082 (0.079, 0.086), SRMS = 0.075], we made some adjustments to the model. First, we excluded BISQ item 5 (average amount of infant nighttime sleep in hours) from the analysis as its loading on the latent variable ‘Infant Night Waking’ was very low (*β* = 0.26). Second, we added four residual correlations between separate items that improved the model fit for the χ^2^ test statistic with at least 80 points: EPDS items 4 and 5 (measuring the mother’s anxiety and feelings of panic), EPDS items 1 and 2 (measuring the mother’s ability to laugh and look forward to enjoyable things), the BNSQ composite variable and BNSQ item 4 (measuring the mother’s daytime sleepiness and average number of night awakenings), and BISQ item 7 and BNSQ item 4 (measuring the average number of night awakenings for the infant, and the average number of night awakenings for the mother). After these adjustments, the fit of the final model indicated adequate fit [χ^2^(127) = 858.445, *P* <0.001, CFI = 0.952, TLI = 0.942, RMSEA = 0.060 (0.056, 0.064), SRMS = 0.059]. The adjusted model is depicted in Fig. 3.

Analyses for the full sample as well as for the subgroups were conducted using the adjusted model. The results are summarized in Table 2.

**Table 2.** Results from the structural regression models

	*n*				*b*	β	95% CI_β_	*SE*_β_	*z*_β_	*P*_β_
Full sample	1598	Direct	BISQ	→ BNSQ	1.10	0.56	[0.48, 0.61]	0.03	16.66	<0.001
		BISQ	→ BF	0.49	0.40	[0.34, 0.46]	0.03	13.34	<0.001
			BNSQ	→ EPDS	0.19	0.55	[0.49, 0.62]	0.03	16.16	<0.001
			BF	→ EPDS	−0.10	−0.17	[−0.24, −0.10]	0.04	−4.97	<0.001
		Indirect	BISQ → BNSQ	→ EPDS	0.21	0.31	[0.25, 0.37]	0.03	9.60	<0.001
			BISQ → BF	→ EPDS	−0.05	−0.07	[−0.10, −0.04]	0.02	−4.57	<0.001
Male infants	853	Direct	BISQ	→ BNSQ	1.02	0.55	[0.47, 0.64]	0.04	12.66	<0.001
		BISQ	→ BF	0.47	0.45	[0.38, 0.53]	0.05	11.68	<0.001
			BNSQ	→ EPDS	0.22	0.59	[0.51, 0.68]	0.05	13.16	<0.001
			BF	→ EPDS	−0.14	−0.22	[−0.32, −0.13]	0.05	−4.68	<0.001
		Indirect	BISQ → BNSQ	→ EPDS	0.22	0.33	[0.24, 0.41]	0.04	7.58	<0.001
			BISQ → BF	→ EPDS	−0.07	−0.10	[−0.15, −0.06]	0.02	−4.29	<0.001
Female infants	745	Direct	BISQ	→ BNSQ	1.22	0.57	[0.48, 0.67]	0.05	11.48	<0.001
		BISQ	→ BF	0.50	0.32	[0.22, 0.42]	0.05	6.52	<0.001
			BNSQ	→ EPDS	0.18	0.51	[0.41, 0.61]	0.05	10.45	<0.001
			BF	→ EPDS	−0.05	−0.11	[−0.20, −0.02]	0.05	−2.45	0.01
		Indirect	BISQ → BNSQ	→ EPDS	0.21	0.29	[0.20, 0.38]	0.05	6.42	<0.001
			BISQ → BF	→ EPDS	−0.03	−0.04	[−0.07, −0.00]	0.02	−2.19	0.03
Using infant formula	1048	Direct	BISQ	→ BNSQ	1.18	0.56	[0.46, 0.64]	0.04	13.33	<0.001
			BISQ	→ BF	0.51	0.39	[0.32, 0.46]	0.03	11.30	<0.001
			BNSQ	→ EPDS	0.17	0.51	[0.43, 0.59]	0.04	12.71	<0.001
			BF	→ EPDS	−0.08	−0.15	[−0.23, −0.07]	0.04	−3.55	<0.001
		Indirect	BISQ → BNSQ	→ EPDS	0.21	0.29	[0.21, 0.36]	0.04	7.59	<0.001
			BISQ → BF	→ EPDS	−0.04	−0.06	[−0.09, −0.02]	0.02	−3.42	0.001
Not using infant formula	524	Direct	BISQ	→ BNSQ	1.21	0.55	[0.44, 0.67]	0.06	9.57	<0.001
			BISQ	→ BF	0.08	0.32	[0.10, 0.55]	0.12	2.80	0.005
			BNSQ	→ EPDS	0.22	0.61	[0.50, 0.71]	0.06	10.81	<0.001
			BF	→ EPDS	−0.24	−0.08	[−0.25, 0.09]	0.09	−0.91	0.36
		Indirect	BISQ → BNSQ	→ EPDS	0.26	0.34	[0.22, 0.44]	0.06	6.13	<0.001
			BISQ → BF	→ EPDS	−0.02	−0.03	[−0.08, 0.03]	0.03	−0.84	0.40
Co−sleeping	259	Direct	BISQ	→ BNSQ	1.46	0.51	[0.35, 0.67]	0.08	6.34	<0.001
			BISQ	→ BF	0.12	0.17	[−0.04, 0.39]	0.11	1.58	0.11
			BNSQ	→ EPDS	0.18	0.58	[0.45, 0.71]	0.07	8.48	<0.001
			BF	→ EPDS	−0.37	−0.28	[−0.48, −0.09]	0.10	−2.86	0.004
		Indirect	BISQ → BNSQ	→ EPDS	0.26	0.30	[0.16, 0.43]	0.07	4.29	<0.001
			BISQ → BF	→ EPDS	−0.04	−0.05	[−0.12, −0.02]	0.04	−1.31	0.19
Sleeping separately	1331	Direct	BISQ	→ BNSQ	1.16	0.57	[0.49, 0.64]	0.04	15.25	<0.001
			BISQ	→ BF	0.52	0.39	[0.33, 0.45]	0.04	11.90	<0.001
			BNSQ	→ EPDS	0.19	0.53	[0.46, 0.60]	0.04	14.57	<0.001
			BF	→ EPDS	−0.09	−0.15	[−0.23, −0.08]	0.04	−4.14	<0.001
		Indirect	BISQ → BNSQ	→ EPDS	0.22	0.30	[0.23, 0.37]	0.03	8.74	<0.001
			BISQ → BF	→ EPDS	−0.04	−0.06	[−0.09, −0.03]	0.02	−3.89	<0.001
Primiparous mothers	826	Direct	BISQ	→ BNSQ	1.04	0.56	[0.48, 0.66]	0.05	12.62	<0.001
		BISQ	→ BF	0.43	0.37	[0.29, 0.45]	0.04	9.42	<0.001
			BNSQ	→ EPDS	0.19	0.52	[0.43, 0.60]	0.04	11.90	<0.001
			BF	→ EPDS	−0.11	−0.19	[−0.28, −0.10]	0.05	−4.21	<0.001
		Indirect	BISQ → BNSQ	→ EPDS	0.19	0.29	[0.21, 0.37]	0.04	7.17	<0.001
			BISQ → BF	→ EPDS	−0.05	−0.07	[−0.11, −0.03]	0.02	−3.81	<0.001
Multiparous mothers	706	Direct	BISQ	→ BNSQ	1.25	0.57	[0.47, 0.68]	0.05	11.19	<0.001
		BISQ	→ BF	0.53	0.39	[0.29, 0.49]	0.05	7.68	<0.001
			BNSQ	→ EPDS	0.19	0.57	[0.46, 0.67]	0.04	10.51	<0.001
			BF	→ EPDS	−0.07	−0.12	[−0.22, −0.02]	0.05	−2.26	0.02
		Indirect	BISQ → BNSQ	→ EPDS	0.24	0.32	[0.22, 0.43]	0.05	6.33	<0.001
			BISQ → BF	→ EPDS	−0.04	−0.05	[−0.09, −0.00]	0.02	−2.12	0.03

BISQ, Brief Infant Sleep Questionnaire; BNSQ, Basic Nordic Sleep Questionnaire; BF, breastfeeding items (indicating breastfeeding duration in months and daily breastfeeding frequency); EPDS, Edinburgh Postnatal Depression Scale. Note that 66 mothers (4.1%) had missing data on the variable measuring parity, and 8 mothers (0.5%) had missing data on the variable measuring co-sleeping.

#### Structural regressions in the full sample

Infant night waking at 6 months postpartum was associated with both increased maternal sleep disturbance (*β* = 0.55) and increased breastfeeding frequency (*β* = 0.40). Maternal sleep disturbance was associated with increased PPD symptoms (*β* = 0.56), and breastfeeding frequency was associated with ‘decreased’ PPD symptoms (*β* = −0.17). Moreover, there was a positive, indirect association between infant night waking and PPD symptoms via maternal sleep disturbance (*β* = 0.31), suggesting that infant night waking, when mediated by maternal sleep disturbance, was associated with increased PPD symptoms. There was also a negative, indirect association between infant night waking and PPD symptoms via breastfeeding frequency (*β* = −0.07), suggesting that infant night waking, when mediated by frequent breastfeeding, in fact, was associated with decreased PPD symptoms.

The directions of the associations remained the same when dividing the participants into the subgroups. However, we noted some slight differences in magnitude of the associations. These differences should however be interpreted with caution, as in each comparison the confidence intervals of the associations overlapped between the two subgroups.

#### Infant’s sex

Noteworthy, the association between infant night waking and breastfeeding frequency as well as the association between maternal sleep disturbance and PPD symptoms were somewhat larger for mothers with male (*β* = 0.45; *β* = 0.59) than female infants (*β* = 0.32; *β* = 0.51). The negative association between breastfeeding frequency and PPD symptoms was also larger for mothers with male (*β* = −0.22) than female infants (*β* = −0.11).

#### Infant formula

The association between maternal sleep disturbance and PPD symptoms was slightly smaller for mothers using infant formula (*β*=0.51) than for mothers not using (*β*=0.61). For mother not using formula, the negative association between breastfeeding frequency and PPD symptoms was not significant (*β*=−0.08, *P*=0.36).

#### Co-sleeping

Mothers who co-slept with their infant reported a slightly larger negative association between breastfeeding frequency and PPD symptoms (*β*=−0.28) than did those who slept separately (*β*=−0.15). The association between infant night waking and breastfeeding frequency was not significant for mothers who co-slept with their infants (*β*=0.17, *P*=0.11).

#### Parity

We noted no major differences between primiparous and multiparous mothers.

## DISCUSSION

In this study, we investigated the association between infant night waking and PPD symptoms at 6 months postpartum. Based on theories regarding evolutionary mother–offspring conflict [14, 17], we expected that infant night waking would be associated with PPD symptoms, and that this association, would, in turn, be mediated by breastfeeding frequency and maternal sleep disturbance.

### Infant night waking and PPD symptoms

In support of our first hypothesis, we found that more frequent infant night waking was associated with higher levels of PPD symptoms at 6 months postpartum. This finding is consistent with previous research, in which infant night waking has been linked to poorer mental and physical health, as well as symptoms of fatigue and depression [30–33]. Another possible explanation for the association between infant night waking and PPD symptoms could be derived from the mismatch hypothesis about recent shifts in human lifestyle [11]. Contrary to the mismatch hypothesis, we did not, however, find any strong support for differences between co-sleepers and mothers who slept separately from their infants (with co-sleeping more closely resembling ancestral lifestyles) [47]. Other potential mismatch factors that contribute to PPD, such as changes in family networks postpartum and observed differences in immune function between women in industrialized environments [48], were not tested here but warrant further investigation. Our theory seeks to explain the potential appearance of PPD symptoms as a consequence of the competition over maternal investment between infant and mother. We view the addition of mother–offspring conflict theory as an extension of other proposed hypotheses on PPD. Indeed, our work is congruent with Hagen’s defection theory [9]—that PPD may be an adaptive response from the mother to seek investment from others (i.e. if the fitness gains of PPD are larger than the costs). In other words, PPD is a ‘counter strategy’ of the mother, which—if successful in such social contexts where it upregulates support—moderates the amount of maternal investment allocated to that offspring. One of the limitations of this study is that it is cross-sectional in design. Future research should study the mother–offspring conflict and PPD through longitudinal designs, as there is also some evidence the mother’s PPD symptoms increase infant night waking [49].

### Maternal sleep disturbance and breastfeeding frequency

In line with our second hypothesis, maternal sleep disturbance mediated the association between infant night waking and PPD symptoms at 6 months postpartum. Worth noting, there is evidence indicating that lower levels of the neuroactive steroid allopregnanolone is associated with both PPD symptoms [50] and lower sleep quality [51], suggesting that poor sleep and PPD might be mechanistically linked (although the causal mechanisms between these are still unclear). Contrary to our third hypothesis, we found a negative association between infant night waking and PPD symptoms at 6 months postpartum, when mediated by breastfeeding frequency. In this case, infant night waking was associated with more frequent breastfeeding, which is in line with previous studies [52]. However, more frequent breastfeeding was associated with ‘less’ PPD symptoms. There are several possible explanations for this association. It is possible that breastfeeding, in fact, protects the mother from developing PPD, e.g. through hormonal changes, such as oxytocin release and decreased cortisol levels [53, 54], through inflammatory modulation [55] (in contrast, PPD has been associated with increased neuroinflammation) [56] and through enhancing bonding and attachment between her and her infant [57]. In other words, the positive effects of breastfeeding might outweigh some negative effects of frequent night waking. Mothers in Nordic countries are often very aware of the benefits of breastfeeding [58]. Consequently, breastfeeding might have a positive effect on maternal mood and physiology. Studies have also linked breastfeeding difficulties, such as pain [59] and low breastfeeding self-efficacy [60], to higher levels of PPD. This may result in self-selection, whereby the mothers that continue to breastfeed are those that feel good about it and therefore show less PPD symptoms. It is also possible that mothers with PPD symptoms breastfeed less. For instance, a recent study of postnatal Malaysian women showed that depressive symptoms were associated with breastfeeding problems, which in turn predicted breastfeeding discontinuation [61]. Finally, there is some evidence that suggests that breastfeeding *per se* does not affect maternal fatigue substantially [62]—in fact, breastfeeding could have a protective effect. The link between PPD, breastfeeding and infant night waking is complex, and this study is not meant to be used to inform medical recommendations on the benefits of breastfeeding, but as evidence to inform evolutionary perspectives on PPD.

### Subgroup analyses

We also explored the associations separately based on the infant’s sex, infant formula use, co-sleeping and parity. We found no significant associations between the subgroups. The association between infant night waking and breastfeeding frequency as well as the association between breastfeeding frequency and PPD symptoms were, however, larger—although not to a statistically significant degree—for mothers with male infants than female. In the literature, there is evidence of milk differences depending on offspring sex across many different mammal taxa [63–65], yet, research on sex-specific milk differences in humans is limited and contradictory [66]. We also noted some trends for infant formula use and co-sleeping. Co-sleepers had a slightly larger negative association between breastfeeding frequency and PPD symptoms, as well as a non-significant association between infant night waking and breastfeeding, both indicating that a potential effect of infant night waking on PPD symptoms would be mediated by fatigue related to lost sleep and not breastfeeding. This is in line with the results of Quillin *et al.* [67], who found that nighttime breastfeeding among co-sleepers was associated with more frequent maternal wakings but greater total sleep duration. However, we did not find support for our prediction that mothers using infant formula would show a weaker effect between infant night waking and PPD symptoms, adding to the inconclusive findings regarding breastfeeding-related fatigue and PPD symptoms. In summary, our current findings suggest there are no large differences in associations depending on infant sex, infant formula use, co-sleeping and parity, and highlight that, if there are differences associated with mother–offspring conflict, their manifestation regarding PPD may be complex.

### Limitations

One of the major limitations of this study was that the included variables were not designed specifically for our research questions. For instance, no item addressed how many times the infant woke during the night specifically to breastfeed. The latent variable for breastfeeding frequency was estimated using items measuring breastfeeding duration in months and the average amount of breastfeeding during a 24-h day, both of which are only proxies of nighttime breastfeeding frequency and the metabolic demand on the mother. Similarly, this study did not distinguish between mothers who experienced sleep disturbance due to pain or discomfort and mothers who woke because of their infant’s wakefulness.

As our study was cross-sectional due to some variables of interest only being available at 6 months postpartum, it is not possible to draw any conclusions about potential causal associations between the factors. Problems before or after 6 months postpartum also remained undetected. It could, for instance, be that the infant’s behavior to extend the interbirth interval starts later than at 6 months postpartum, due to the suppressed fertility within the first months postpartum regardless of breastfeeding pattern [23]. All measurements used in this study were retrospective self-report questionnaires, which allows mothers to over- or underestimate her or the infant’s behavior. A more reliable method to gather information about infant night waking could be, e.g. through real time motion-based measurements. Moreover, PPD symptoms could also affect the experiences by the mothers, for instance so that mothers with PPD symptoms would be more sensitive to infant night waking, for instance due to pre-existing sleeping problems. Future studies could also gain from measuring prenatal baseline rates of PPD symptoms.

It is also worth noting that our sample consisted only of mothers living in Finland, limiting the generalizability of the results. Compared to other high-income countries, the Nordic countries stand out with more generous paid parental leave policies [68]—possibly contributing to lower levels of fatigue compared to non-Nordic mothers. The prevalence of co-sleeping in our sample (16%) was also considerably lower than in, for instance, many East Asian and African countries [69]. In addition, forms of peri- and postnatal social support might differ considerably between cultures, with, for instance, grandmaternal support being associated with higher maternal peri- and postnatal health in Himba women [10]. Previous research also highlights a link between PPD and ‘lacking’ social support [12], indicating that the relationship between these two aspects might be complex, and that PPD does not necessarily result in higher levels of negotiated social support (i.e. contrasting the defection theory). Interestingly, a recent study of postnatal Hadza foragers did not find an association between PPD symptoms and social support [13]. However, the authors noted an association between sleep disturbance and PPD symptoms. Future work should more closely examine whether PPD actually leads to increased social support, as correlational evidence is insufficient for drawing causal conclusions.

## CONCLUSIONS AND IMPLICATIONS

To the best of our knowledge, this study is the first to investigate the association between infant night waking, breastfeeding frequency, maternal sleep disturbance and PPD symptoms based on theoretical assumptions of evolutionary mother–offspring conflict. The results supported our first hypothesis that infant night waking is associated with PPD symptoms. Moreover, the association between infant night waking and PPD symptoms was mediated by maternal sleep disturbance. Breastfeeding frequency, however, was associated with fewer PPD symptoms.

Although the results support some of the theoretical predictions regarding mother–offspring conflict, additional research regarding the consequences of infant night waking is warranted. In particular, longitudinal designs would provide more information about potential causal relationships between infant night waking, maternal fatigue and PPD symptoms. Future studies would also benefit from including information about breast pumping, which caregiver is performing night feedings (with bottles/formula), antidepressant use, and other factors that might affect the relationship between infant night waking, breastfeeding and maternal fatigue. Future research should also investigate whether infant night waking, indeed, contributes to longer interbirth intervals; as such an investigation would further test the theoretical assumption regarding mother–offspring conflict. Studying PPD symptoms from the perspective of evolutionary conflict between the mother and her offspring would improve our understanding of the involved behavioral processes of both parties, and thus allow interventions designed to improve individual well-being and support the mother–offspring relationship.

## Supplementary data

Supplementary data is available at *EMPH* online.

## FUNDING 

This work was supported by the Academy of Finland (grant number 298513 to J.A.), and the Sundell Foundation (J.A.).

**Conflict of interest:** None declared. 

## Supplementary Material

eoaa049_Supplementary_Data

## References

[1] American Psychiatric Association. Diagnostic and Statistical Manual of Mental Disorders. 5th edn. Washington: American Psychiatric Association, 2013.

[2] O'Hara MW , McCabe JE. Postpartum depression: current status and future directions. Annu Rev Clin Psychol 2013;9:379–407. 23394227 10.1146/annurev-clinpsy-050212-185612

[3] Gavin N , Gaynes B, Lohr K et al Perinatal depression: a systematic review of prevalence and incidence. Obstet Gynecol 2005;106:1071–83. 16260528 10.1097/01.AOG.0000183597.31630.db

[4] O'Hara M , Swain A. Rates and risk of postpartum depression – a meta-analysis. Int Rev Psychiatry 1996;8:37–54.

[5] Shorey S , Chee CY, Ng ED et al Prevalence and incidence of postpartum depression among healthy mothers: a systematic review and meta-analysis. J Psychiatr Res 2018;104:235–48. 30114665 10.1016/j.jpsychires.2018.08.001

[6] Slomian J , Honvo G, Emonts P et al Consequences of maternal postpartum depression: a systematic review of maternal and infant outcomes. Womens Health 2019;15:174550651984404. 10.1177/1745506519844044 PMC6492376 31035856

[7] Chen YH , Tsai SY, Lin HC. Increased mortality risk among offspring of mothers with postnatal depression: a nationwide population-based study in Taiwan. Psychol Med 2011;41:2287–96. 21524332 10.1017/S0033291711000584

[8] Weobong B , ten Asbroek AH, Soremekun S et al Association between probable postnatal depression and increased infant mortality and morbidity: findings from the DON population-based cohort study in rural Ghana. BMJ Open 2015;5:e006509. 10.1136/bmjopen-2014-006509 PMC4554911 26316646

[9] Hagen E. The functions of postpartum depression. Evo Hum Behav 1999;20:325–59.

[10] Scelza BA , Hinde K. Crucial contributions. Hum Nat 2019;30:371–97. 31802396 10.1007/s12110-019-09356-2 PMC6911617

[11] Hahn-Holbrook J , Haselton M. Is postpartum depression a disease of modern civilization? Curr Dir Psychol Sci 2014;23:395–400. 28503034 10.1177/0963721414547736 PMC5426853

[12] Yim IS , Stapleton LR, Guardino CM et al Biological and psychosocial predictors of postpartum depression: systematic review and call for integration. Annu Rev Clin Psychol 2015;11:99–137. 25822344 10.1146/annurev-clinpsy-101414-020426 PMC5659274

[13] Herlosky KN , Benyshek DC, Mabulla IA et al Postpartum maternal mood among Hadza foragers of Tanzania: a mixed methods approach. Cult Med Psychiatry 2020;44:305–32. 31646409 10.1007/s11013-019-09655-4

[14] Black MM , Baqui AH, Zaman K et al Depressive symptoms among rural Bangladeshi mothers: implications for infant development. J Child Psychol Psychiatry 2007;48:764–72. 17683448 10.1111/j.1469-7610.2007.01752.x

[15] Correa H , Castro e Couto T, Santos W et al Postpartum depression symptoms among Amazonian and Northeast Brazilian women. J Affect Disord 2016;204:214–8. 27372408 10.1016/j.jad.2016.06.026

[16] Glasser S , Stoski E, Kneler V et al Postpartum depression among Israeli Bedouin women. Arch Womens Ment Health 2011;14:203–8. 21479760 10.1007/s00737-011-0216-4

[17] Trivers RL. Parental investment and sexual selection. In: Campbell B (ed.). Sexual Selection and the Descent of Man, 1871-1971. Chicago: Aldine, 1972, 136–79.

[18] Hamilton WD. The genetical evolution of social behaviour I. J Theor Biol 1964;7:1–16. 5875341 10.1016/0022-5193(64)90038-4

[19] Conde-Agudelo A , Rosas-Bermúdez A, Kafury-Goeta AC. Birth spacing and risk of adverse perinatal outcomes: a meta-analysis. JAMA 2006;295:1809–23. 16622143 10.1001/jama.295.15.1809

[20] Blurton Jones NGB , da Costa E. A suggested adaptive value of toddler night waking: delaying the birth of the next sibling. Ethol Sociobiol 1987;8:135–42.

[21] Marlowe FW. Hunter-gatherers and human evolution. Evol Anthropol 2005;14:54–67. 10.1016/j.jhevol.2014.03.006 24746602

[22] Kotler J , Haig D. The tempo of human childhood: a maternal foot on the accelerator, a paternal foot on the brake. Evol Anthropol 2018;27:80–91. 29575348 10.1002/evan.21579 PMC5947556

[23] Haig D. Troubled sleep: night waking, breastfeeding and parent-offspring conflict. Evol Med Public Health 2014;2014:32–9. 24610432 10.1093/emph/eou005 PMC3982900

[24] Thapa S , Short R, Potts M. Breast feeding, birth spacing and their effects on child survival. Nature 1988;335:679–82. 3173491 10.1038/335679a0

[25] Howie P , McNeilly A. Effect of breast-feeding patterns on human birth intervals. Reproduction 1982;65:545–57. 10.1530/jrf.0.0650545 7097656

[26] Martin MA , Garcia G, Kaplan HS et al Conflict or congruence? Maternal and infant-centric factors associated with shorter exclusive breastfeeding durations among the Tsimane. Soc Sci Med 2016;170:9–17. 27732906 10.1016/j.socscimed.2016.10.003 PMC5107317

[27] Brown A , Harries V. Infant sleep and night feeding patterns during later infancy: association with breastfeeding frequency, daytime complementary food intake, and infant weight. Breastfeed Med 2015;10:246–52. 25973527 10.1089/bfm.2014.0153

[28] De Judicibus MA , McCabe MP. Psychological factors and the sexuality of pregnant and postpartum women. J Sex Res 2002;39:94–103. 12476241 10.1080/00224490209552128

[29] White ND. Influence of sleep on fertility in women. Am J Lifestyle Med 2016;10:239–41. 30202278 10.1177/1559827616641386 PMC6125064

[30] Bayer J , Hiscock H, Hampton A et al Sleep problems in young infants and maternal mental and physical health. J Paediatr Child Health 2007;43:66–73. 17207059 10.1111/j.1440-1754.2007.01005.x

[31] Giallo R , Rose N, Vittorino R. Fatigue, wellbeing and parenting in mothers of infants and toddlers with sleep problems. J Reprod Infant Psychol 2011;29:236–49.

[32] Meltzer L , Mindell J. Relationship between child sleep disturbances and maternal sleep, mood, and parenting stress: a pilot study. J Fam Psychol 2007;21:67–73. 17371111 10.1037/0893-3200.21.1.67

[33] Wilson N , Lee JJ, Bei B. Postpartum fatigue and depression: a systematic review and meta-analysis. J Affect Disord 2019;246:224–33. 30584956 10.1016/j.jad.2018.12.032

[34] Wake M , Morton-Allen E, Poulakis Z et al Prevalence, stability, and outcomes of cry-fuss and sleep problems in the first 2 years of life: prospective community-based study. Pediatrics 2006;117:836–42. 16510665 10.1542/peds.2005-0775

[35] Valeggia C , Ellison P. Interactions between metabolic and reproductive functions in the resumption of postpartum fecundity. Am J Hum Biol 2009;21:559–66. 19298003 10.1002/ajhb.20907 PMC3305908

[36] Richter D , Krämer M, Tang N et al Long-term effects of pregnancy and childbirth on sleep satisfaction and duration of first-time and experienced mothers and fathers. Sleep 2019;42:zsz015. 30649536 10.1093/sleep/zsz015

[37] Pietikäinen JT , Polo-Kantola P, Pölkki P et al Sleeping problems during pregnancy—a risk factor for postnatal depressiveness. Arch Womens Ment Health 2019;22:327–37. 30121844 10.1007/s00737-018-0903-5

[38] Hiscock H , Wake M. Infant sleep problems and postnatal depression: a community-based study. Pediatrics 2001;107:1317–22. 11389250 10.1542/peds.107.6.1317

[39] Karlsson L , Tolvanen M, Scheinin NM et al; FinnBrain Birth Cohort Study Group. Cohort profile: the FinnBrain birth cohort study (FinnBrain). Int J Epidemiol 2018;47:15–6j. 29025073 10.1093/ije/dyx173

[40] Meltzer LJ , Mindell JA. Sleep and sleep disorders in children and adolescents. Psychiatr Clin 2006;29:1059–76. 10.1016/j.psc.2006.08.004 17118282

[41] Sadeh A. A brief screening questionnaire for infant sleep problems: validation and findings for an internet sample. Pediatrics 2004;113:e570–77. 15173539 10.1542/peds.113.6.e570

[42] Huang YS , Paiva T, Hsu JF et al Sleep and breathing in premature infants at 6 months post-natal age. BMC Pediatr 2014;14:303. 25510740 10.1186/s12887-014-0303-6 PMC4272529

[43] Partinen M , Gislason T. Basic Nordic Sleep Questionnaire (BNSQ): a quantitated measure of subjective sleep complaints. J Sleep Res 1995;4:150–5. 10.1111/j.1365-2869.1995.tb00205.x 10607192

[44] Cox J , Holden J, Sagovsky R. Detection of postnatal depression: development of the 10-item Edinburgh Postnatal Depression Scale. Brit J Psychiat 1987;150:782–6. 10.1192/bjp.150.6.782 3651732

[45] Gibson J , McKenzie-McHarg K, Shakespeare J et al A systematic review of studies validating the Edinburgh Postnatal Depression Scale in antepartum and postpartum women. Acta Psychiatr Scand 2009;119:350–64. 19298573 10.1111/j.1600-0447.2009.01363.x

[46] Jadresic E , Araya R, Jara C. Validation of the Edinburgh Postnatal Depression Scale (EPDS) in Chilean postpartum women. J Psychosom Obstet Gynecol 1995;16:187–91. 10.3109/01674829509024468 8748993

[47] McKenna JJ , Ball HL, Gettler LT. Mother–infant cosleeping, breastfeeding and sudden infant death syndrome: what biological anthropology has discovered about normal infant sleep and pediatric sleep medicine. Am J Phys Anthropol 2007;134:133–61. 10.1002/ajpa.20736 18046747

[48] Natri H , Garcia AR, Buetow KH et al The pregnancy pickle: evolved immune compensation due to pregnancy underlies sex differences in human diseases. Trends Genet 2019;35:478–88. 31200807 10.1016/j.tig.2019.04.008 PMC6611699

[49] Warren SL , Howe G, Simmens SJ et al Maternal depressive symptoms and child sleep: models of mutual influence over time. Dev Psychopathol 2006;18:1–16. 16478549 10.1017/S0954579406060019

[50] Osborne LM , Gispen F, Sanyal A et al Lower allopregnanolone during pregnancy predicts postpartum depression: an exploratory study. Psychoneuroendocrinology 2017;79:116–21. 28278440 10.1016/j.psyneuen.2017.02.012 PMC5420429

[51] Terán-Pérez G , Arana-Lechuga Y, Esqueda-León E et al Steroid hormones and sleep regulation. Mini Rev Med Chem 2012;12:1040–8. 23092405 10.2174/138955712802762167

[52] DeLeon C , Karraker K. Intrinsic and extrinsic factors associated with night waking in 9-month-old infants. Infant Behav Dev 2007;30:596–605. 17416420 10.1016/j.infbeh.2007.03.009

[53] Abou-Saleh MT , Ghubash R, Karim L et al Hormonal aspects of postpartum depression. Psychoneuroendocrinology 1998; 23:465–75. 9802121 10.1016/s0306-4530(98)00022-5

[54] Moberg KU , Prime DK. Oxytocin effects in mothers and infants during breastfeeding. Infant 2013;9:201–6.

[55] Zimmer JP , Garza C, Butte NF et al Maternal blood B‐cell (CD19+) percentages and serum immunoglobulin concentrations correlate with breast‐feeding behavior and serum prolactin concentration. Am J Reprod Immunol 1998;40:57–62. 9689362 10.1111/j.1600-0897.1998.tb00389.x

[56] Anderson G , Maes M. Postpartum depression: psychoneuroimmunological underpinnings and treatment. Neuropsychiatr Dis Treat 2013;9:277–87. 23459664 10.2147/NDT.S25320 PMC3582478

[57] Gibbs B , Forste R, Lybbert E. Breastfeeding, parenting, and infant attachment behaviors. Matern Child Health J 2018;22:579–88. 29388115 10.1007/s10995-018-2427-z

[58] Laanterä S , Pölkki T, Ekström A et al Breastfeeding attitudes of Finnish parents during pregnancy. BMC Pregnancy Childbirth 2010;10:79. 21126368 10.1186/1471-2393-10-79 PMC3003624

[59] Watkins S , Meltzer-Brody S, Zolnoun D et al Early breastfeeding experiences and postpartum depression. Obstet Gynecol 2011;118:214–21. 21734617 10.1097/AOG.0b013e3182260a2d

[60] Haga S , Ulleberg P, Slinning K et al A longitudinal study of postpartum depressive symptoms: multilevel growth curve analyses of emotion regulation strategies, breastfeeding self-efficacy, and social support. Arch Womens Ment Health 2012;15:175–84. 22451329 10.1007/s00737-012-0274-2

[61] Dib S , Fewtrell M, Wells JC et al The influence of hospital practices and family support on breastfeeding duration, adverse events, and postnatal depression among first-time mothers. Malaysian J Med Health Sci 2020;16:90–8.

[62] Callahan S , Séjourné N, Denis A. Fatigue and breastfeeding: an inevitable partnership? J Hum Lact 2006;22:182–7. 16684906 10.1177/0890334406286972

[63] Hinde K , Foster AB, Landis LM et al Daughter dearest: sex‐biased calcium in mother's milk among rhesus macaques. Am J Phys Anthropol 2013;151:144–50. 23446791 10.1002/ajpa.22229

[64] Hinde K , Carpenter AJ, Clay JS et al Holsteins favor heifers, not bulls: biased milk production programmed during pregnancy as a function of fetal sex. PLoS One 2014;9:e86169. 24498270 10.1371/journal.pone.0086169 PMC3911898

[65] Landete-Castillejos T , García A, López-Serrano FR et al Maternal quality and differences in milk production and composition for male and female Iberian red deer calves (Cervus elaphus hispanicus). Behav Ecol Sociobiol 2005;57:267–74.

[66] Galante L , Milan AM, Reynolds CM et al Sex-specific human milk composition: the role of infant sex in determining early life nutrition. Nutrients 2018;10:1194. 10.3390/nu10091194 PMC6165076 30200404

[67] Quillin SI , Glenn LL. Interaction between feeding method and co‐sleeping on maternal‐newborn sleep. J Obstet Gynecol Neonatal Nurs 2004;33:580–8. 10.1177/0884217504269013 15495703

[68] Ray R , Gornick JC, Schmitt J. Who cares? Assessing generosity and gender equality in parental leave policy designs in 21 countries. J Eur Soc Policy 2010;20:196–216.

[69] Mileva-Seitz VR , Bakermans-Kranenburg MJ, Battaini C, Luijk MP. Parent-child bed-sharing: the good, the bad, and the burden of evidence. Sleep Med Rev 2017;32:4–27. 27107752 10.1016/j.smrv.2016.03.003

