# Supplementary material for: Postpartum depression and mother–offspring conflict over maternal investment
Source: Evol Med Public Health. 2021 Jan 2;9(1):11–23. doi: 10.1093/emph/eoaa049 (PMC7910802; doi:10.1093/emph/eoaa049)
Supplement: eoaa049_Supplementary_Data [file eoaa049_supplementary_data.docx]

| Online Supplementary Table 1  *Descriptive Statistics for the Study Variables* | | | | | | |
| --- | --- | --- | --- | --- | --- | --- |
| Factor | Scale | Item | Description | *M* | *SD* | min–max |
| Infant night waking | BISQ | 5^a^ | Nighttime sleep in hours | 9.93 | 1.18 | 1–18 |
|  |  | 7 | Night awakenings | 2.43 | 1.77 | 0–15 |
|  |  | 8 | Hours spent awake during the night | 0.45 | 0.48 | 0–5 |
|  |  | 12 | Sleep problems | 0.30 | 0.49 | 0–2 |
| Maternal sleep disturbance | BNSQ | 4 | Night awakenings | 3.37 | 1.03 | 1–5 |
|  |  | 12^a^ | Nighttime sleep in hours | 7.45 | 1.11 | 2–12 |
|  |  | Sleep | Daytime sleepiness (composite variable) | 8.27 | 2.59 | 4–20 |
| Breastfeeding frequency |  | Month | Breastfeeding duration in months | 4.36 | 1.66 | 0–6 |
|  |  | Day | Breastfeeding frequency during a 24-hour day | 3.39 | 3.79 | 0–16 |
| Postpartum depression symptoms | EPDS | 1 | Able to laugh and see the funny side of things | 0.23 | 0.47 | 0–3 |
|  |  | 2 | Looked forward with enjoyment to things | 0.19 | 0.48 | 0–3 |
|  |  | 3 | Blamed myself unnecessarily when things went wrong | 0.93 | 0.86 | 0–3 |
|  |  | 4 | Anxious or worried for no good reason | 0.62 | 0.80 | 0–3 |
|  |  | 5 | Felt scared or panicky for no good reason | 0.33 | 0.66 | 0–3 |
|  |  | 6 | Things have been getting on top of me | 0.80 | 0.76 | 0–3 |
|  |  | 7 | So unhappy that I have had difficulty sleeping | 0.29 | 0.59 | 0–3 |
|  |  | 8 | Felt sad or miserable | 0.67 | 0.68 | 0–3 |
|  |  | 9 | So unhappy that I have been crying | 0.36 | 0.57 | 0–3 |
|  |  | 10 | The thought of harming myself has occurred to me | 0.06 | 0.30 | 0–3 |
| *Note.* BISQ = Brief Infant Sleep Questionnaire; BNSQ = Basic Nordic Sleep Questionnaire; EPDS = Edinburgh Postnatal Depression Scale. Higher scores indicate more infant night waking, maternal sleep disturbance, breastfeeding, and postpartum depression symptoms at six months postpartum. ^a^ For clarity, scores for these items are here presented before reversing. | | | | | | |
